# Supplementary material for: New Evidence for the Role of the Blood-Brain Barrier and Inflammation in Stress-Associated Depression: A Gene-Environment Analysis Covering 19,296 Genes in 109,360 Humans
Source: Int J Mol Sci. 2024 Oct 21;25(20):11332. doi: 10.3390/ijms252011332 (PMC11508422; doi:10.3390/ijms252011332)
Supplement: Supplementary file 1 [file ijms-25-11332-s001.zip › Blood-brain barrier in stress-associated depression supplementary materials_v4.pdf]

Supplementary Materials for

New evidence for the role of the blood-brain barrier and inflammation in stress-associated depression: a gene-environment analysis covering 19,296 genes in 109,360 humans

Zsofia Gal[1,2], Dora Torok[1,2], Xenia Gonda[2,3], Nora Eszlari[1,2], Ian Muir Anderson[4], John Francis William Deakin[4], Peter Petschner[1,2,5,6], Gabriella Juhasz[1,2,†], Gyorgy Bagdy[1,2,†,*]

[1] Department of Pharmacodynamics, Faculty of Pharmaceutical Sciences, Semmelweis University, 1089, Budapest, Hungary

[2] NAP3.0-SE Neuropsychopharmacology Research Group, Hungarian Brain Research Program, Semmelweis University, 1089, Budapest, Hungary

[3] Department of Psychiatry and Psychotherapy, Semmelweis University, 1083, Budapest, Hungary

[4] Neuroscience and Psychiatry Unit, Division of Neuroscience and Experimental Psychology, School of Biological Sciences, Faculty of Biological, Medical and Human Sciences, The University of Manchester and Manchester Academic Health Sciences Centre, M13 9NT, Manchester, United Kingdom

[5] Bioinformatics Center, Institute of Chemical Research, Kyoto University, 611-0011, Uji, Kyoto, Japan

[6] Research Unit for Realization of Sustainable Society, Kyoto University, Gokasho, 611-0011, Uji, Kyoto, Japan

[*] Correspondence: bagdy.gyorgy@semmelweis.hu; Gyorgy Bagdy, PhD, DSc, Department of Pharmacodynamics, Faculty of Pharmaceutical Sciences, Semmelweis University

[†] These authors contributed equally to this work.

Table of contents

Supplementary Table S1. - Population characteristics of the UK Biobank cohort. 3
Supplementary Figure S1. Correlation plot for PHQ9 depression mean values and adult traumatic events scores in UKB cohort..4
Supplementary Figure S2. Manhattan plot of SNPs from GWEIS on PHQ9 depressive symptoms mean value in interaction with adult stressors in the whole UKB cohort 5
Supplementary Figure S3. Manhattan plot of SNPs in male subjects of UKB from GWEIS on PHQ9 depressive symptoms mean value in interaction with adult stressors 6
Supplementary Figure S4. Manhattan plot of SNPs in female subjects of UKB from GWEIS on PHQ9 depressive symptoms mean value in interaction with adult stressors 7
Supplementary Figure S5. Manhattan plot of genes from GWEIS on PHQ9 depressive symptoms mean value in interaction with adult stressors in UKB 8
Chi square statistics with BBB-related genes, based on human postmortem microvascular structures from the temporal lobe 9
Supplementary Figure S6. Manhattan plot of gene-based GWEIS on PHQ9 depression using adult traumatic events as interaction factor in UKB cohort...9
Supplementary Figure S7. Venn-diagram of significant gene-level results..10
Supplementary Figure S8. Manhattan plot of genes in male subjects of UKB from GWEIS on PHQ9 depressive symptoms mean value in interaction with adult stressors 11
Supplementary Figure S9. Manhattan plot of genes in female subjects of UKB from GWEIS on PHQ9 depressive symptoms mean value in interaction with adult stressors 12
Chi square test of inflammatory-related genes in men and women 12
Supplementary Table S11. Population characteristics of the NewMood cohort..13
Supplementary Figure S10. Manhattan plot of SNPs from GWEIS on BSI depressive symptoms mean value in interaction with last year's stressors score in the whole NM cohort...14
Supplementary Figure S11. Manhattan plot of genes from GWEIS on BSI depressive symptoms mean value in interaction with last year's stressors score in the whole NM cohort...15
Supplementary Figure S12. Manhattan plot of SNPs in male subjects of NM from GWEIS on BSI depressive symptoms mean value in interaction with last year's stressors score 16
Supplementary Figure S13. Manhattan plot of genes in male subjects of NM from GWEIS on BSI depressive symptoms mean value in interaction with last year's stressors score 17
Supplementary Figure S14. Manhattan plot of SNPs in female subjects of NM from GWEIS on BSI depressive symptoms mean value in interaction with last year's stressors score 18
Supplementary Figure S15. Manhattan plot of genes in female subjects of NM on BSI depressive symptoms mean value in interaction with last year's stressors score...19
Supplementary Figure S16. Replication of the 17 BBB-related significant genes of the UKB GWEIS analysis in NM...20
Supplementary Figure S17. Replication of the 23 inflammatory-related significant genes of the UKB GWEIS analysis in NM 21
Results of sign test in male- and female subgroups separately 22
Supplementary Figure S18. Replication of the 13 BBB-related significant genes in male subjects of the UKB analysis in NM 22
Supplementary Figure S19. Replication of the 10 inflammatory-related significant genes of the UKB GWEIS analysis in male subjects of the NM 23
Supplementary Figure S20. Replication of the 14 BBB-related significant genes in female subjects of the UKB analysis in NM...24
Supplementary Figure S21. Replication of the 11 inflammatory-related significant genes in female subjects of the UKB analysis in NM 25
References...26

Population characteristics of the UK Biobank cohort

**all participants**					
	**mean**	**SD**	**min**	**max**	**n**
**age**	56.19	7.67	39	72	109,360
**PHQ9 depression**	1.31	0.41	1	4	109,360
**ATE stress**	0.40	0.50	0	4	109,360
**male subjects**					
	**mean**	**SD**	**min**	**max**	**n**
**age**	56.84	7.72	39	72	48,277
**PHQ9 depression**	1.27	0.39	1	4	48,277
**ATE stress**	0.34	0.40	0	4	48,277
**female subjects**					
	**mean**	**SD**	**min**	**max**	**n**
**age**	55.66	7.59	40	70	61,083
**PHQ9 depression**	1.34	0.42	1	4	61,083
**ATE stress**	0.48	0.57	0	4	61,083

Supplementary Table S1. - Population characteristics of the UK Biobank cohort. *n - number of subjects provided information on the given variable; min - minimum value of the given variable; max - maximum value of the given variable; mean - mean value of the given variable; SD - standard deviation of the given variable; PHQ9 depression - mean values of depression scores based on PHQ9 depression questionnaire in UKB; ATE stress - mean values of adult stress events in UKB*

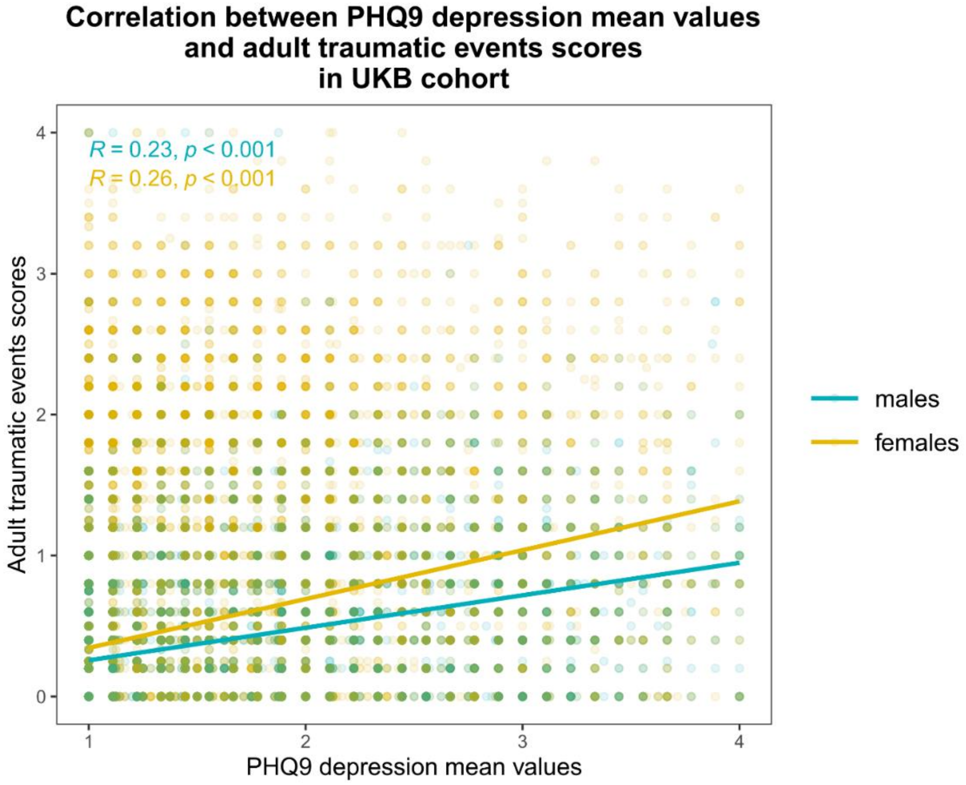

Supplementary Figure S1. Correlation plot for PHQ9 depression mean values and adult traumatic events scores in UKB cohort, with correlation coefficients for male- and female subjects separately. In the combined UKB cohort of male- and female subjects, Pearson's correlation coefficient R for PHQ9 depression values and adult traumatic events scores was 0.26 ($p < 0.001$). A week, but statistically significant correlation can be detected between PHQ9 depression mean values and adult traumatic events score. The correlation coefficient is smaller in men (R = 0.23), than in women (R = 0.26). The causality of correlation cannot be determined.

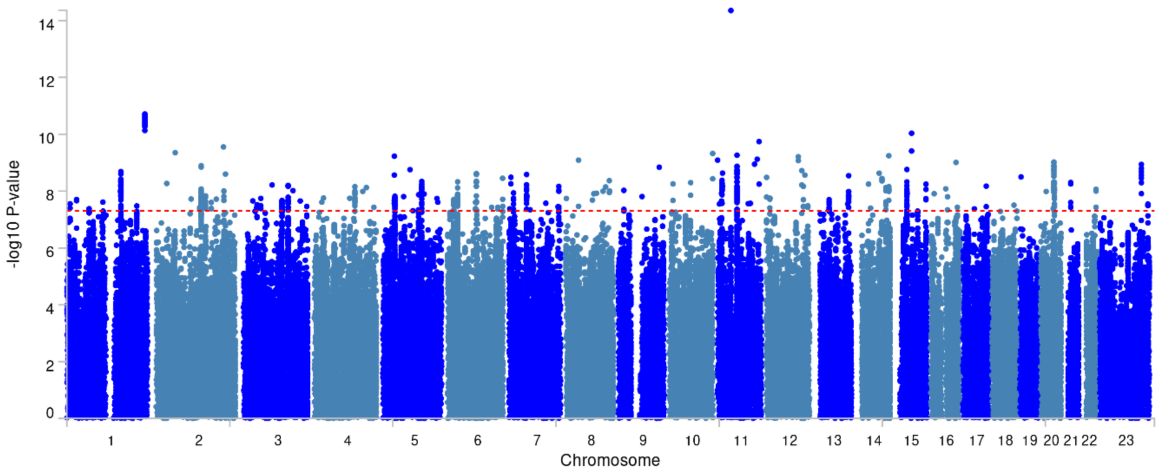

Supplementary Figure S2. Manhattan plot of SNPs from GWEIS on PHQ9 depressive symptoms mean value in interaction with adult stressors in the whole UKB cohort. The x-axis represents the chromosomal location of genetic variants across the genome (each chromosome is shown sequentially from chr1 to chr23). The y-axis represents the -log10 of the p-values for the interaction between each genetic variant and adult traumatic events score on PHQ9 depression mean values. The horizontal red line indicates the genome-wide significance threshold ($p = 5 * 10^{-8}$). Points above this line denote genetic variants with significant interaction effects (n = 788). The most significant SNP, rs117435652 ($\beta = 0.0636$; $p = 4.4 * 10^{-15}$) is an intron variant of *LRRC4C* gene, positioned on chromosome 11.

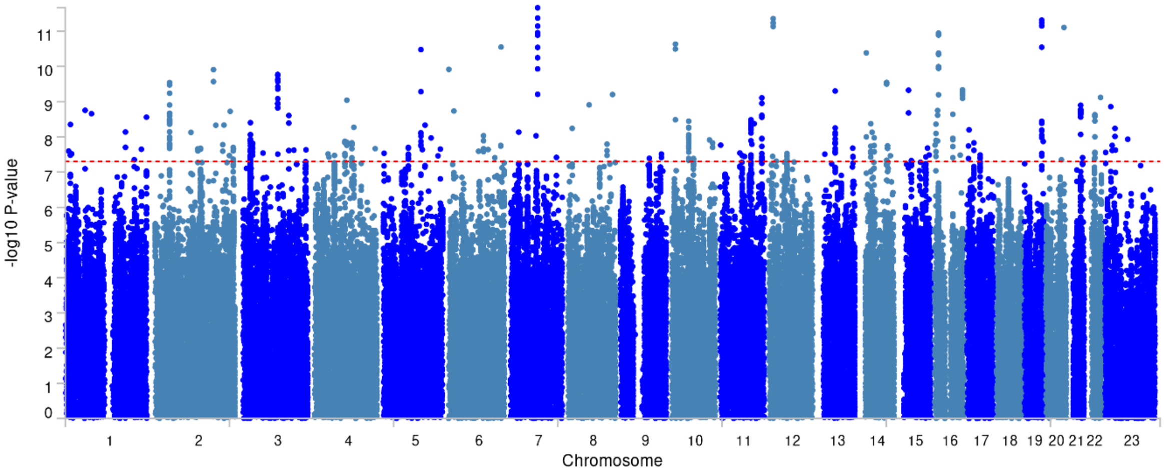

Supplementary Figure S3. Manhattan plot of SNPs in male subjects of UKB from GWEIS on PHQ9 depressive symptoms mean value in interaction with adult stressors. The x-axis represents the chromosomal location of genetic variants across the genome (each chromosome is shown sequentially from chr1 to chr23). The y-axis represents the -log10 of the p-values for the interaction between each genetic variant and adult traumatic events score on PHQ9 depression mean values in male subjects of the UKB cohort. The horizontal red line indicates the genome-wide significance threshold ($p = 5 * 10^{-8}$). Points above this line denote genetic variants with significant interaction effects (n = 412). The most significant SNP, rs74297459 ($\beta = 0.0265$; $p = 2.15 * 10^{-12}$) is an intergenic variant on chromosome 7.

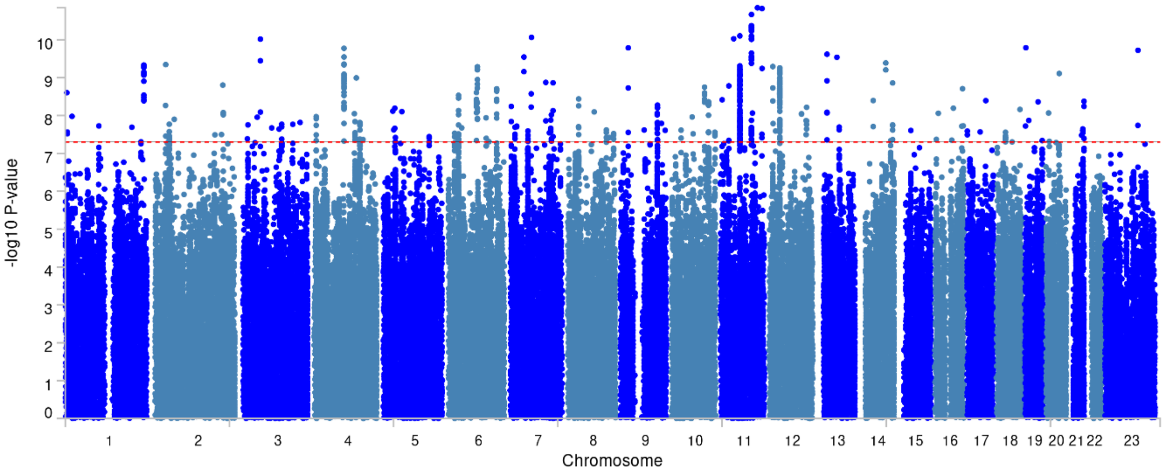

Supplementary Figure S4. Manhattan plot of SNPs in female subjects of UKB from GWEIS on PHQ9 depressive symptoms mean value in interaction with adult stressors. The x-axis represents the chromosomal location of genetic variants across the genome (each chromosome is shown sequentially from chr1 to chr23). The y-axis represents the -log10 of the p-values for the interaction between each genetic variant and adult traumatic events score on PHQ9 depression mean values in female subjects of the UKB cohort. The horizontal red line indicates the genome-wide significance threshold ($p = 5 * 10^{-8}$). Points above this line denote genetic variants with significant interaction effects ($n = 631$). The most significant SNP, rs76262850 ($\beta = 0.0763$; $p = 1.43 * 10^{-11}$) is a regulatory region variant on chromosome 11.

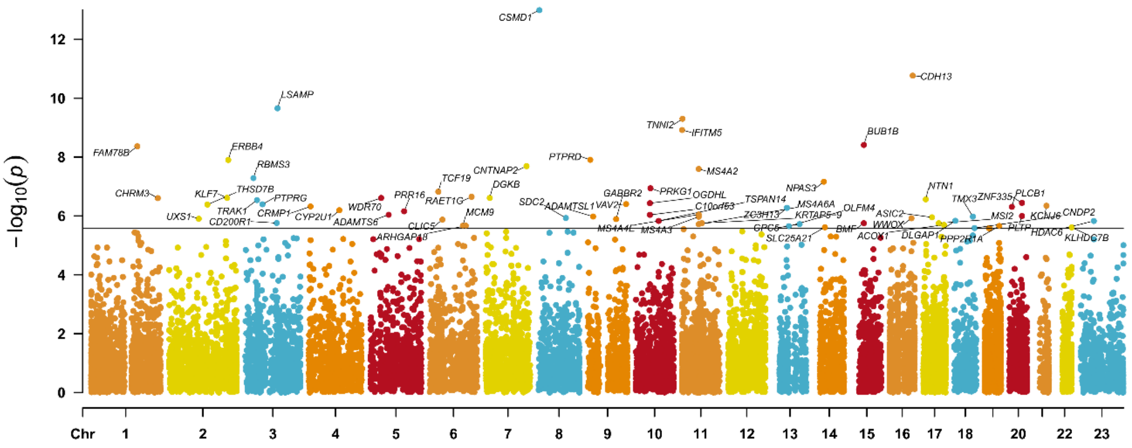

Supplementary Figure S5. Manhattan plot of genes from GWEIS on PHQ9 depressive symptoms mean value in interaction with adult stressors in UKB. The x-axis represents the chromosomal location of genes across the genome (each chromosome is shown sequentially from chr1 to chr23). The y-axis represents the -log10 of the gene-based p-values, converted from the interaction between each genetic variant in a gene and adult traumatic events score on PHQ9 depression mean values in the UKB cohort. The horizontal black line indicates the gene-level genome-wide significance threshold ($p = 2.591 * 10^{-6}$). Points above this line denote genes with significant interaction effects ($n = 63$). The most significant gene was *CSMD1* (Z stat = 7.3461; $p = 1.02 * 10^{-13}$) on chromosome 8.

Chi square statistics with BBB-related genes, based on human postmortem microvascular structures from the temporal lobe

In order to reinforce our findings on enrichment of BBB-related genes among the significant results of GWEIS on PHQ9 depression in interaction with adult stressors, we replicated the chi square statistics using different gene expression data as reference [1]. In this dataset, 3389 genes were considered as BBB-related (from that, 30 remained significant after correction for multiple testing (Supplementary Figure S6)), compared to Puvogel et al.'s study [2], where 1364 genes were detected as highly expressed in different types of cells of BBB. The results, with Pearson's $\chi 2$ (1, n = 19,296) = 39.31; p = $3.62*10^{-10}$ strengthened the findings of BBB-enrichment among the significant results with 2.71-times increase.

In case of men, 23 genes could be connected to BBB, from 44 significant genes: Pearson's $\chi 2$ (1, n = 19,296) = 36.61; p = $1.44*10^{-9}$. In women, 21 from 45 significant genes were considered as BBB-related: Pearson's $\chi 2$ (1, n = 19,296) = 26.33; p = $2.89*10^{-7}$.

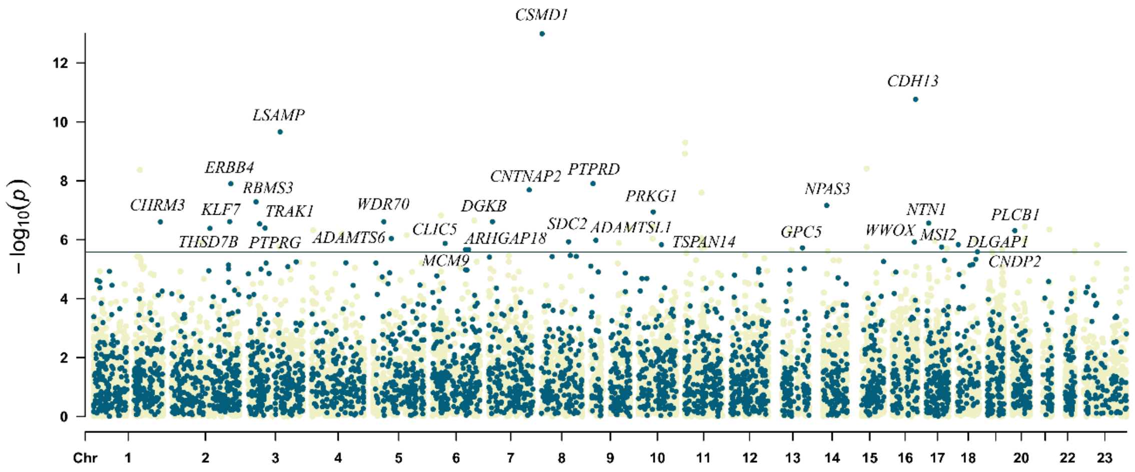

Supplementary Figure S6. Manhattan plot of gene-based GWEIS on PHQ9 depression using adult traumatic events as interaction factor in UKB cohort. The x-axis represents the chromosomal location of genes across the genome (each chromosome is shown sequentially from chr1 to chr23). The y-axis represents the -log10 of the gene-based p-values, converted from the interaction between each genetic variant in a gene and adult traumatic events score on PHQ9 depression mean values in the UKB cohort. The horizontal black line indicates the gene-level genome-wide significance threshold (p = $2.591 * 10^{-6}$). Genes related to BBB, based on gene expression data [1] are highlighted with blue. Points above this line denote genes with significant interaction effects (n = 63). Points above this line, highlighted with blue denote BBB-related significant genes (n = 30). The most significant gene was *CSMD1* (Z stat = 7.3461; p = $1.02 * 10^{-13}$) on chromosome 8.

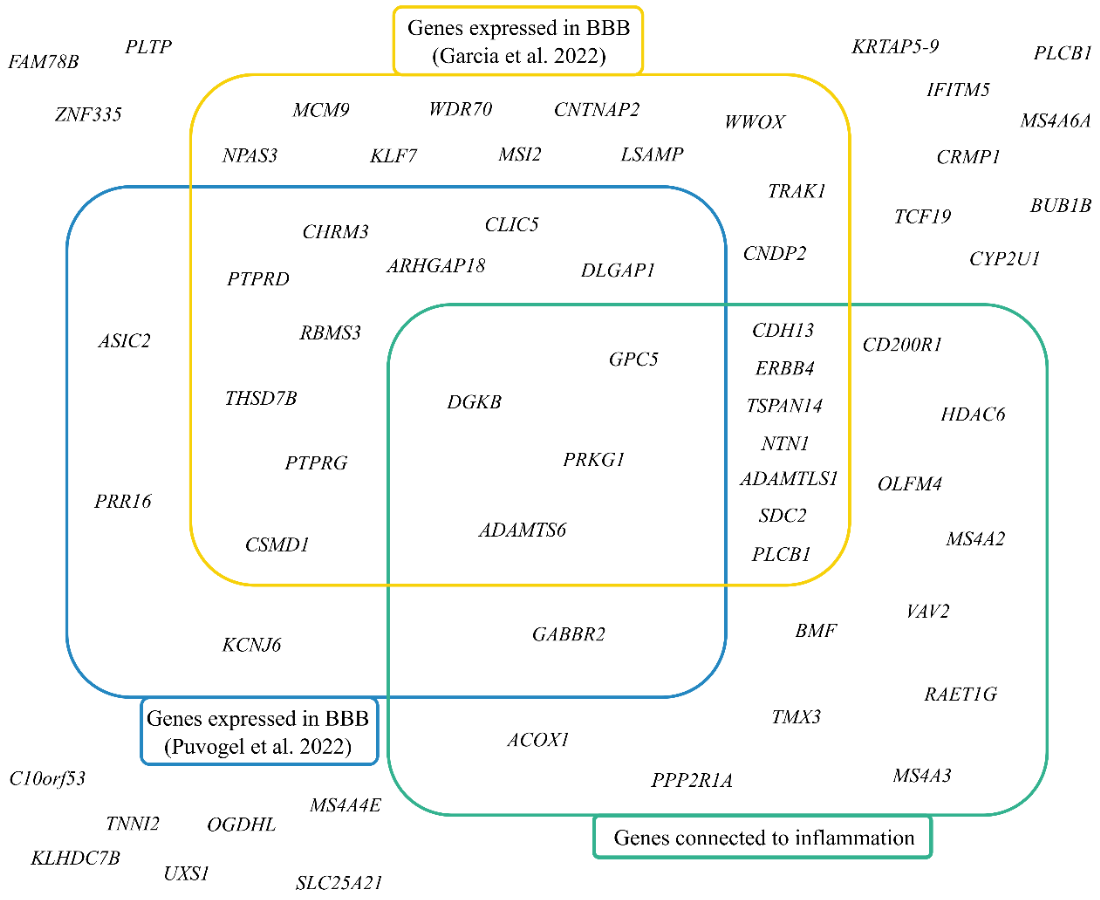

Supplementary Figure S7. Venn-diagram of significant gene-level results. BBB associated genes are based and marked by two different papers, according to a shorter list from Puvogel et al. 2022 [2], and a longer list by Garcia et al. 2022 [1]; genes connected to inflammatory processes are based on MSigDB inflammatory-related gene sets. From 63 significant genes, 23 genes can be considered as inflammatory-related; 17 genes were shown to be expressed in human midbrain BBB cells; 30 genes were shown to be expressed in human postmortem temporal lobe microvasculature.

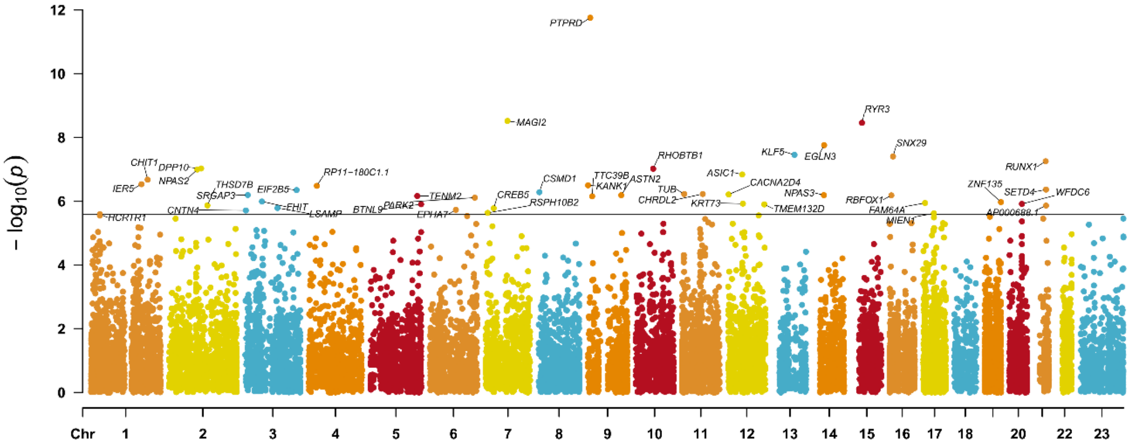

Supplementary Figure S8. Manhattan plot of genes in male subjects of UKB from GWEIS on PHQ9 depressive symptoms mean value in interaction with adult stressors. The x-axis represents the chromosomal location of genes across the genome (each chromosome is shown sequentially from chr1 to chr23). The y-axis represents the -log10 of the gene-based p-values, converted from the interaction between each genetic variant in a gene and adult traumatic events score on PHQ9 depression mean values in male subjects of UKB cohort. The horizontal black line indicates the gene-level genome-wide significance threshold ($p = 2.591 * 10^{-6}$). Points above this line denote genes with significant interaction effects ($n = 44$). The most significant gene was *PTPRD* (Z stat = 6.9549; $p = 1.76 * 10^{-12}$) on chromosome 9.

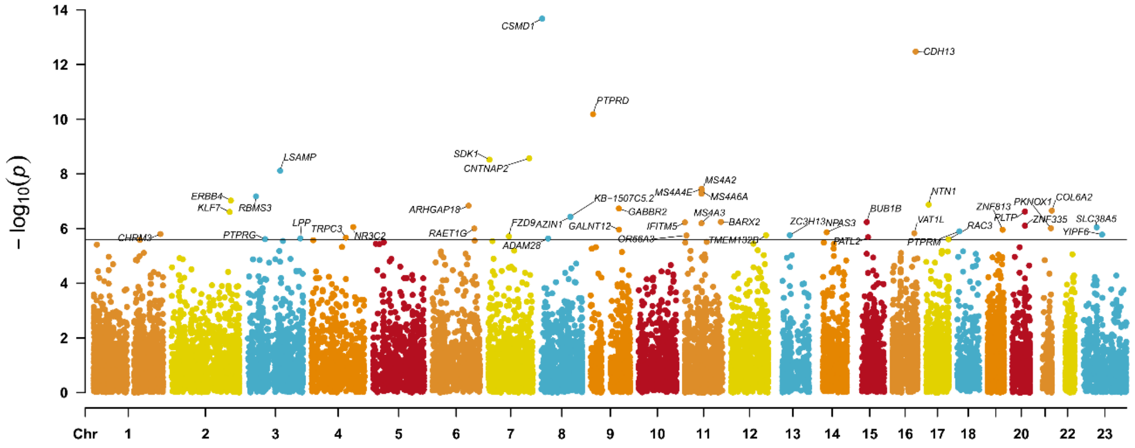

Supplementary Figure S9. Manhattan plot of genes in female subjects of UKB from GWEIS on PHQ9 depressive symptoms mean value in interaction with adult stressors. The x-axis represents the chromosomal location of genes across the genome (each chromosome is shown sequentially from chr1 to chr23). The y-axis represents the -log10 of the gene-based p-values, converted from the interaction between each genetic variant in a gene and adult traumatic events score on PHQ9 depression mean values in female subjects of UKB cohort. The horizontal black line indicates the gene-level genome-wide significance threshold ($p = 2.591 * 10^{-6}$). Points above this line denote genes with significant interaction effects (n = 45). The most significant gene was *CSMD1* (Z stat = 7.5556; $p = 2.08 * 10^{-14}$) on chromosome 8.

Chi square test of inflammatory-related genes in men and women

Pearson's $\chi2$ of inflammatory-related genes among the significant results in men: (1, n = 19.296) = 0.011; p = 0.977.

Pearson's $\chi2$ of inflammatory-related genes among the significant results in women: (1, n = 19.296) = 0.06; p = 0.806.

Population characteristics of the NewMood cohort

all participants					
	**mean**	**SD**	**min**	**max**	**n**
**age**	32.56	10.49	18	60	1,753
**BSI depression**	0.84	0.92	0	4	1,753
**recent stress**	1.21	1.27	0	8	1,753
**male subjects**					
	**mean**	**SD**	**min**	**max**	**n**
**age**	34.15	10.18	18	60	501
**BSI depression**	0.68	0.82	0	4	501
**recent stress**	1.07	1.25	0	8	501
**female subjects**					
	**mean**	**SD**	**min**	**max**	**n**
**age**	31.92	10.54	18	60	1,252
**BSI depression**	0.91	0.95	0	4	1,252
**recent stress**	1.26	1.28	0	7	1,252

Supplementary Table S11. Population characteristics of the NewMood cohort. *n - number of subjects provided information on the given variable; min - minimum value of the given variable; max - maximum value of the given variable; mean - mean value of the given variable; SD - standard deviation of the given variable; BSI depression - mean values of depression scores based on Brief Symptom Inventory in NM cohort; recent stress - recent stress scores in the previous year in NM cohort*

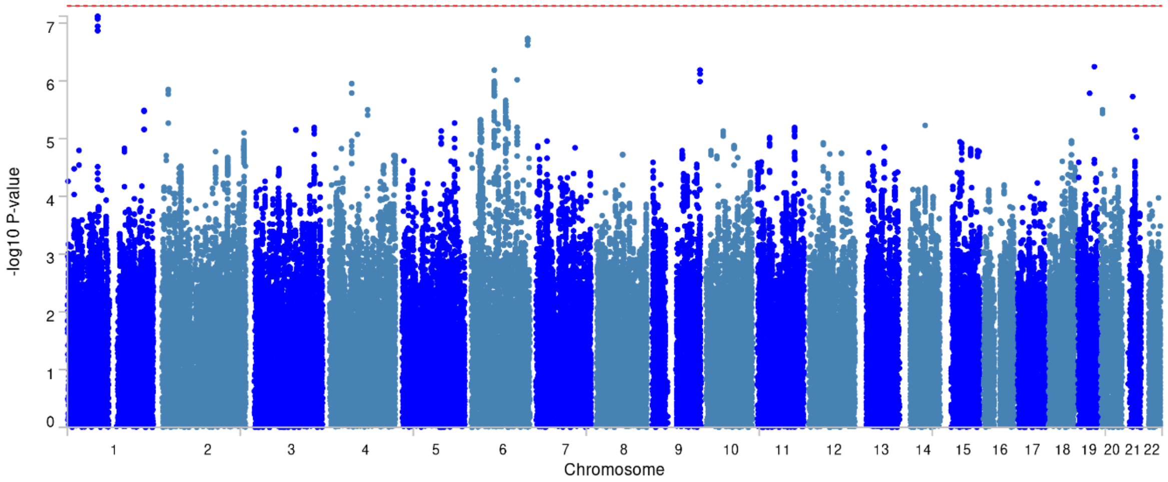

Supplementary Figure S10. Manhattan plot of SNPs from GWEIS on BSI depressive symptoms mean value in interaction with last year's stressors score in the whole NM cohort. The x-axis represents the chromosomal location of genetic variants across the genome (each chromosome is shown sequentially from chr1 to chr22). The y-axis represents the -log10 of the p-values for the interaction between each genetic variant and last 2-year's stressors score on BSI depression mean values. The horizontal red line indicates the genome-wide significance threshold ($p = 5 * 10^{-8}$). None of the SNPs survived correction for multiple testing.

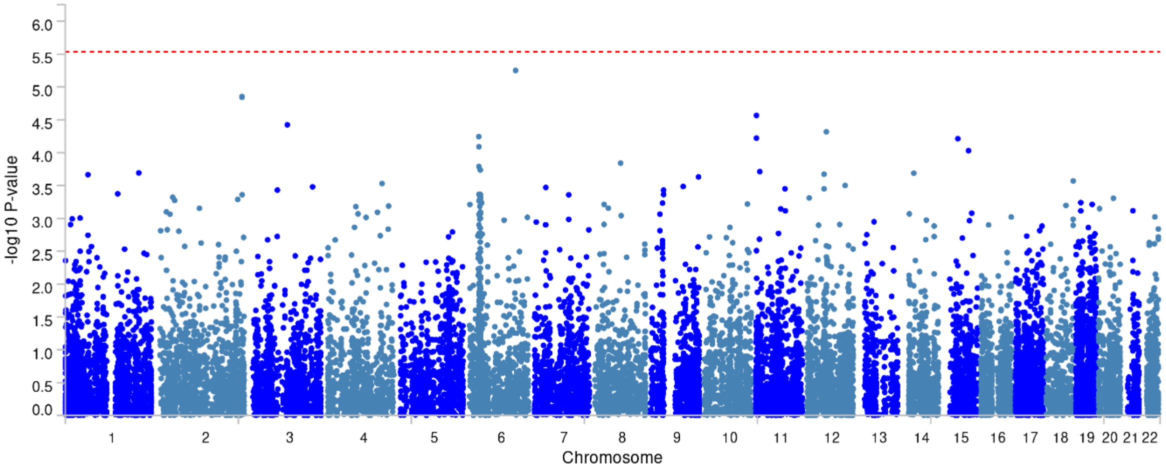

Supplementary Figure S11. Manhattan plot of genes from GWEIS on BSI depressive symptoms mean value in interaction with last year's stressors score in the whole NM cohort. The x-axis represents the chromosomal location of genes across the genome (each chromosome is shown sequentially from chr1 to chr22). The y-axis represents the -log10 of the gene-based p-values, converted from the interaction between each genetic variant in a gene and last 2-years's stressors score on BSI depression mean values in the whole NM cohort. The horizontal red line indicates the gene-level genome-wide significance threshold ($p = 2.895 * 10^{-6}$). No gene survived correction for multiple testing.

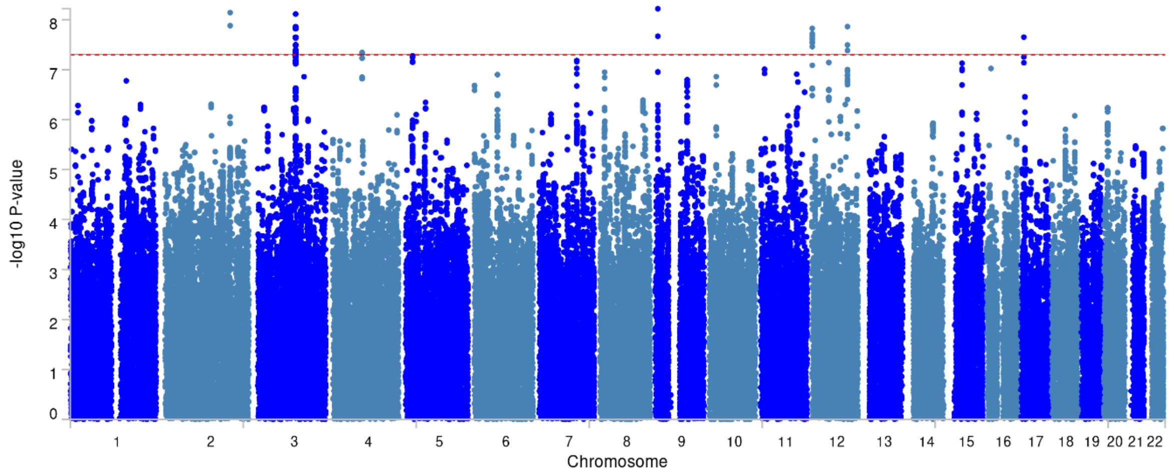

Supplementary Figure S12. Manhattan plot of SNPs in male subjects of NM from GWEIS on BSI depressive symptoms mean value in interaction with last year's stressors score. The x-axis represents the chromosomal location of genetic variants across the genome (each chromosome is shown sequentially from chr1 to chr22). The y-axis represents the -log10 of the p-values for the interaction between each genetic variant and last 2-year's stressors score on BSI depression mean values in male subjects of NM cohort. The horizontal red line indicates the genome-wide significance threshold ($p = 5 * 10^{-8}$). Points above this line denote genetic variants with significant interaction effects (n = 71). The most significant SNP, rs115172658 ($\beta = -0.3614$; $p = 6.06 * 10^{-9}$) is an intron variant of *KDM4C* gene on chromosome 9.

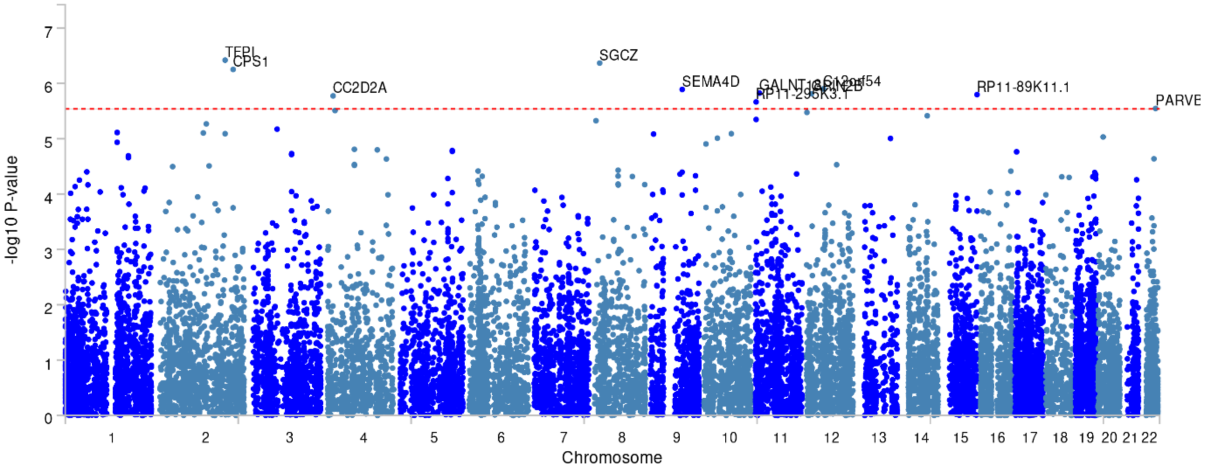

Supplementary Figure S13. Manhattan plot of genes in male subjects of NM from GWEIS on BSI depressive symptoms mean value in interaction with last year's stressors score. The x-axis represents the chromosomal location of genes across the genome (each chromosome is shown sequentially from chr1 to chr22). The y-axis represents the -log10 of the gene-based p-values, converted from the interaction between each genetic variant in a gene and last 2-years's stressors score on BSI depression mean values in male subjects of the NM cohort. The horizontal red line indicates the gene-level genome-wide significance threshold ($p = 2.895 * 10^{-6}$). Points above this line denote genes with significant interaction effects (n = 11). The most significant gene was *TFPI* (Z stat = 4.9445; $p = 3.82 * 10^{-7}$) on chromosome 2.

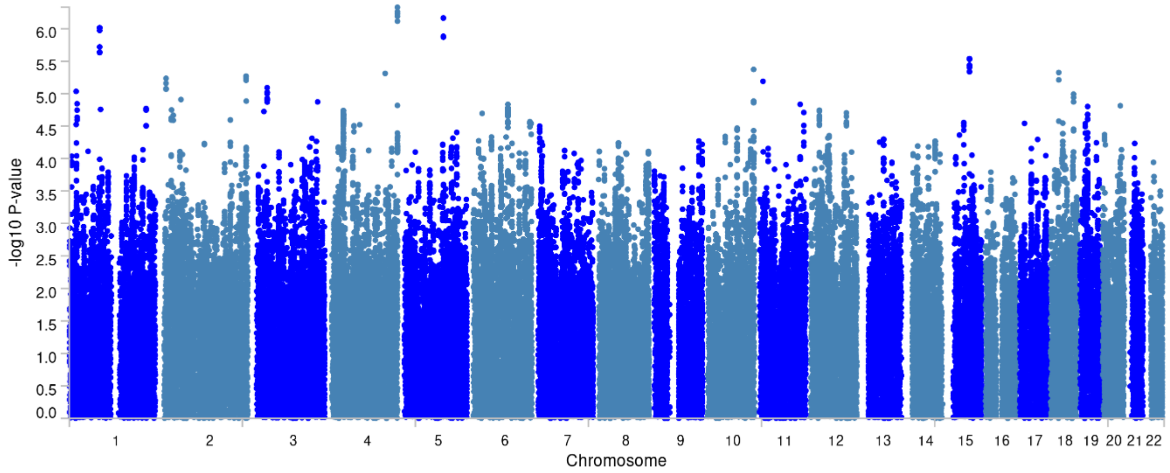

Supplementary Figure S14. Manhattan plot of SNPs in female subjects of NM from GWEIS on BSI depressive symptoms mean value in interaction with last year's stressors score. The x-axis represents the chromosomal location of genetic variants across the genome (each chromosome is shown sequentially from chr1 to chr22). The y-axis represents the -log10 of the p-values for the interaction between each genetic variant and last 2-year's stressors score on BSI depression mean values in female subjects of NM cohort. None of the SNPs survived correction for multiple testing ($p = 5 * 10^{-8}$).

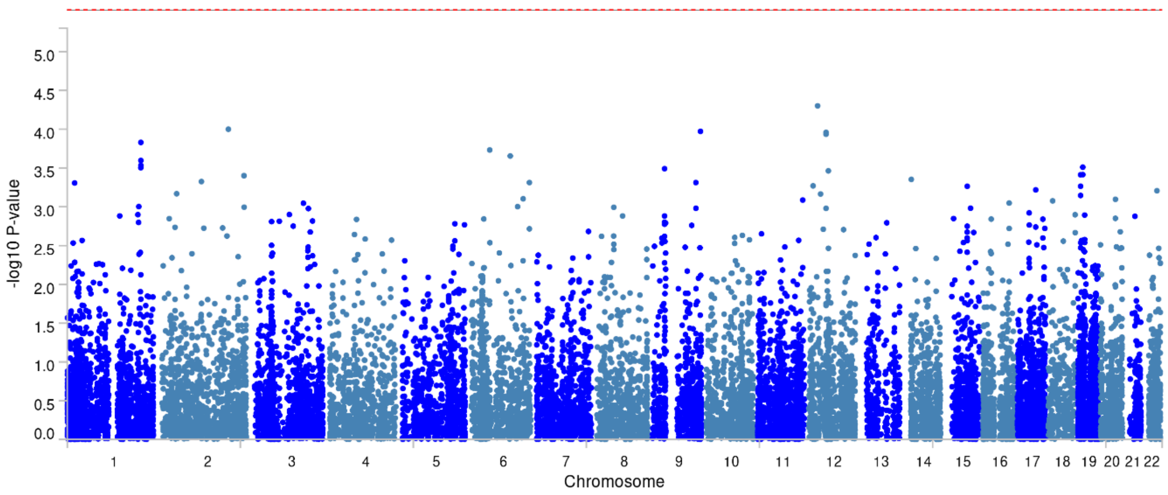

Supplementary Figure S15. Manhattan plot of genes in female subjects of NM on BSI depressive symptoms mean value in interaction with last year's stressors score. The x-axis represents the chromosomal location of genes across the genome (each chromosome is shown sequentially from chr1 to chr22). The y-axis represents the -log10 of the gene-based p-values, converted from the interaction between each genetic variant in a gene and last 2-years's stressors score on BSI depression mean values in female subjects of the NM cohort. The horizontal red line indicates the gene-level genome-wide significance threshold (p = 2.895 * 10^{-6}). No genes survived correction for multiple testing.

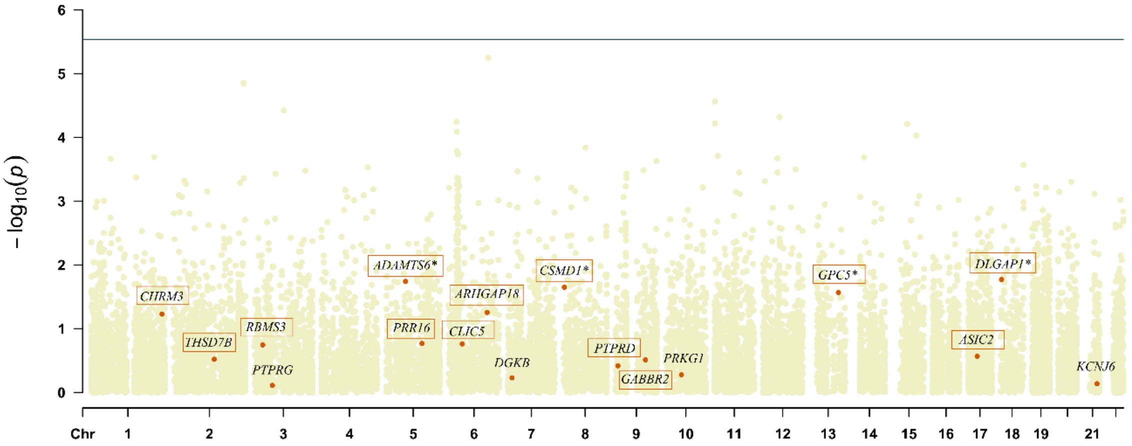

Supplementary Figure S16. Replication of the 17 BBB-related significant genes of the UKB GWEIS analysis in NM. The x-axis represents the chromosomal location of genes across the genome (each chromosome is shown sequentially from chr1 to chr22). The y-axis represents the -log10 of the gene-based p-values, converted from the interaction between each genetic variant in a gene and last 2-years's stressors score on BSI depression mean values in the whole NM cohort. The horizontal black line indicates the gene-level genome-wide significance threshold ($p = 2.895 * 10^{-6}$). No gene survived correction for multiple testing. The 17 BBB-related genes, which remained significant after correction for multiple testing in the gene-level GWEIS conducted in the whole UKB cohort on PHQ9 depression mean values in interaction with adult traumatic events scores, are marked with orange and their symbols. From the 17 BBB-related genes, which were significant in the GWEIS on the whole UKB cohort, genes with nominal significance ($p < 0.05$) in the NM cohort are marked with an asterisk (n = 4); genes with identical Z statistics' (positive) sign (n = 13) in the NM cohort are framed.

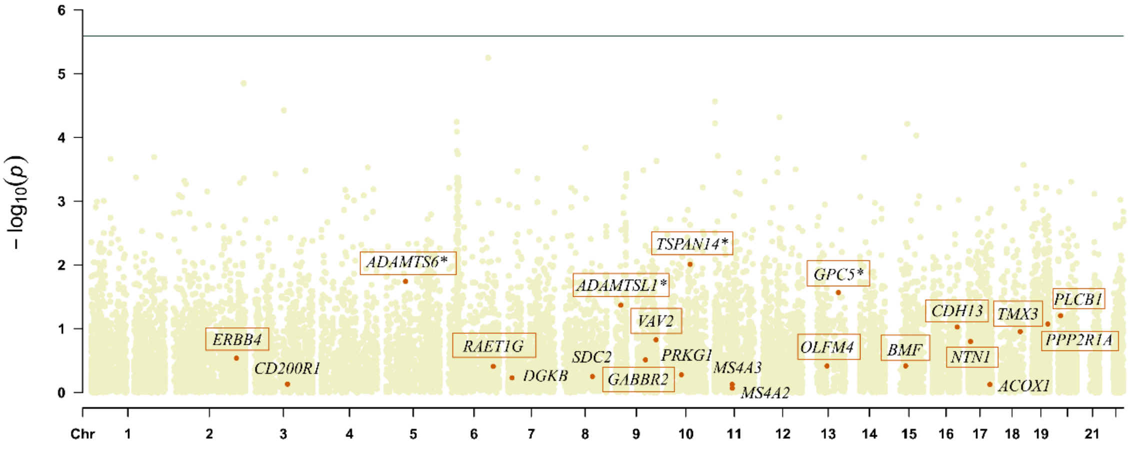

Supplementary Figure S17. Replication of the 23 inflammatory-related significant genes of the UKB GWEIS analysis in NM. The x-axis represents the chromosomal location of genes across the genome (each chromosome is shown sequentially from chr1 to chr22). The y-axis represents the -log10 of the gene-based p-values, converted from the interaction between each genetic variant in a gene and last 2-years's stressors score on BSI depression mean values in the whole NM cohort. The horizontal black line indicates the gene-level genome-wide significance threshold ($p = 2.895 * 10^{-6}$). No gene survived correction for multiple testing. The 23 inflammatory-related genes, which remained significant after correction for multiple testing in the gene-level GWEIS conducted in the whole UKB cohort on PHQ9 depression mean values in interaction with adult traumatic events scores, are marked with orange and their symbols. From the 23 inflammatory-related genes, which were significant in the GWEIS on the whole UKB cohort, genes with nominal significance ($p < 0.05$) in the NM cohort are marked with an asterisk (n = 4); genes with identical Z statistics' (positive) sign (n = 15) in the NM cohort are framed.

Results of sign test in male- and female subgroups separately

In case of male subjects, the sign test showed 12 identically positive Z statistics' scores in the NM cohort as a replication of 13 BBB-related genes based on gene expression data [2], which were significant in the GWEIS analysis in UKB on PHQ9 depressive values in interaction with adult stress scores in male subjects (Supplementary Figure S18.).

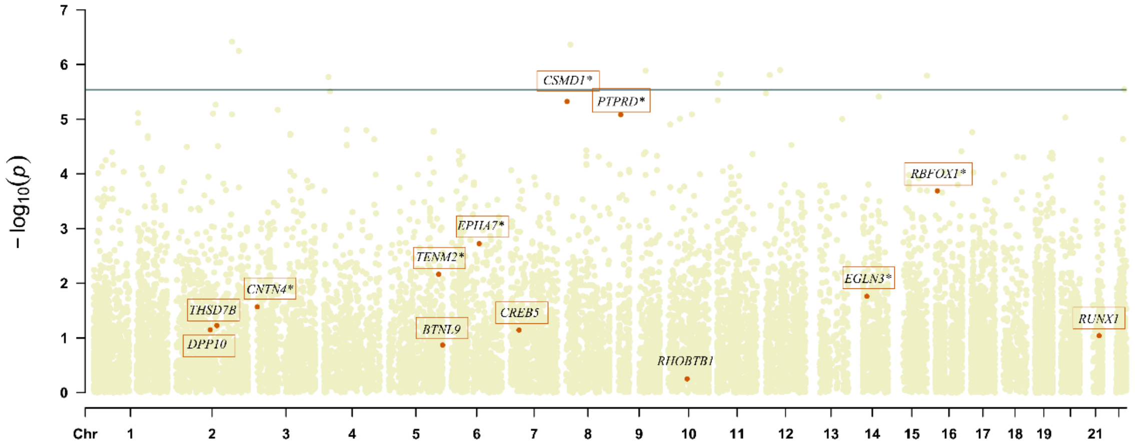

Supplementary Figure S18. Replication of the 13 BBB-related significant genes in male subjects of the UKB analysis in NM. The x-axis represents the chromosomal location of genes across the genome (each chromosome is shown sequentially from chr1 to chr22). The y-axis represents the -log10 of the gene-based p-values, converted from the interaction between each genetic variant in a gene and last 2-years's stressors score on BSI depression mean values in male subjects NM cohort. The horizontal black line indicates the gene-level genome-wide significance threshold ($p = 2.895 * 10^{-6}$). The 13 BBB-related genes, which remained significant after correction for multiple testing in the gene-level GWEIS conducted in male subjects of UKB cohort on PHQ9 depression mean values in interaction with adult traumatic events scores, are marked with orange and their symbols. From the 13 BBB-related genes, which were significant in the GWEIS in male subjects of UKB cohort, genes with nominal significance ($p < 0.05$) in the NM cohort are marked with an asterisk ($n = 7$); genes with identical Z statistics' (positive) sign ($n = 12$) in the NM cohort are framed.

In case of inflammatory-related genes, the sign test showed 7 genes, which were significant in the GWEIS analysis in UKB on PHQ9 depressive values in interaction with adult stress scores in male subjects (Supplementary Figure S19.).

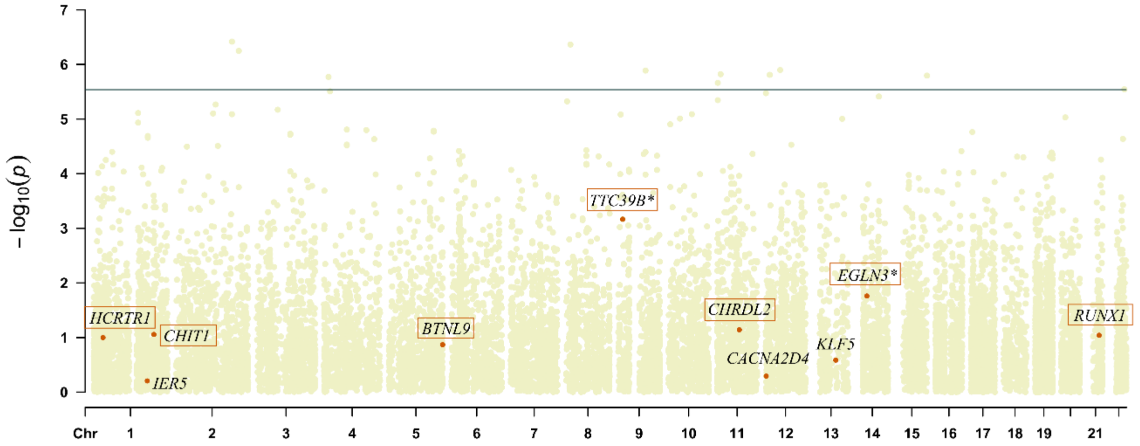

Supplementary Figure S19. Replication of the 10 inflammatory-related significant genes of the UKB GWEIS analysis in male subjects of the NM. The x-axis represents the chromosomal location of genes across the genome (each chromosome is shown sequentially from chr1 to chr22). The y-axis represents the -log10 of the gene-based p-values, converted from the interaction between each genetic variant in a gene and last 2-years's stressors score on BSI depression mean values in male subjects NM cohort. The horizontal black line indicates the gene-level genome-wide significance threshold (p = $2.895 * 10^{-6}$). The 10 inflammatory-related genes, which remained significant after correction for multiple testing in the gene-level GWEIS conducted in male subjects of UKB cohort on PHQ9 depression mean values in interaction with adult traumatic events scores, are marked with orange and their symbols. From the 10 inflammatory-related genes, which were significant in the GWEIS in male subjects of UKB cohort, genes with nominal significance ($p < 0.05$) in the NM cohort are marked with an asterisk (n = 2); genes with identical Z statistics' (positive) sign (n = 7) in the NM cohort are framed.

In female subjects, the sign test - as a replication of the 14 significant gene-based, BBB-related GWEIS results of the UKB cohort using the NM population - resulted in 9 genes with identically positive Z statistics' score (Supplementary Figure S20.).

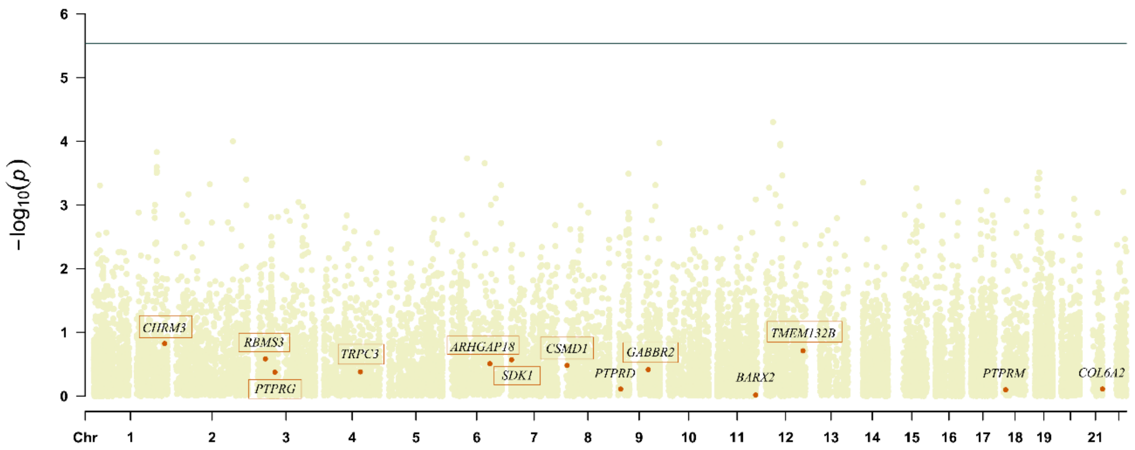

Supplementary Figure S20. Replication of the 14 BBB-related significant genes in female subjects of the UKB analysis in NM. The x-axis represents the chromosomal location of genes across the genome (each chromosome is shown sequentially from chr1 to chr22). The y-axis represents the -log10 of the gene-based p-values, converted from the interaction between each genetic variant in a gene and last 2-years's stressors score on BSI depression mean values in female subjects of NM cohort. The horizontal black line indicates the gene-level genome-wide significance threshold ($p = 2.895 * 10^{-6}$). The 14 BBB-related genes, which remained significant after correction for multiple testing in the gene-level GWEIS conducted in female subjects of UKB cohort on PHQ9 depression mean values in interaction with adult traumatic events scores, are marked with orange and their symbols. From the 14 BBB-related genes, which were significant in the GWEIS in female subjects of UKB cohort, genes with identical Z statistics' (positive) sign (n = 9) in the NM cohort are framed.

In female subjects, the sign test - as a replication of the 11 significant gene-based, inflammatory-related GWEIS results of the UKB cohort using the NM population - resulted in 6 genes with identically positive Z statistics' score (Supplementary Figure S21.).

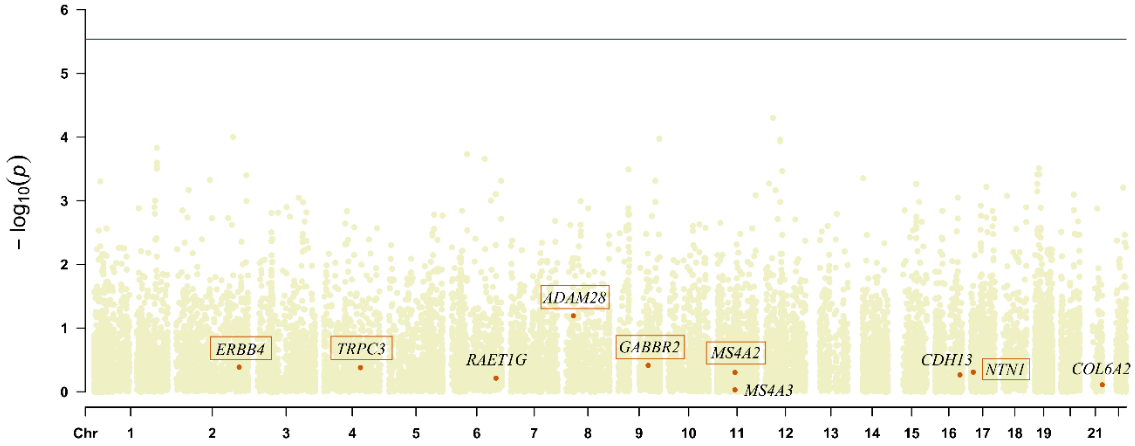

Supplementary Figure S21. Replication of the 11 inflammatory-related significant genes in female subjects of the UKB analysis in NM. The x-axis represents the chromosomal location of genes across the genome (each chromosome is shown sequentially from chr1 to chr22). The y-axis represents the -log10 of the gene-based p-values, converted from the interaction between each genetic variant in a gene and last 2-years's stressors score on BSI depression mean values in female subjects of NM cohort. The horizontal black line indicates the gene-level genome-wide significance threshold ($p = 2.895 * 10^{-6}$). The 11 inflammatory-related genes, which remained significant after correction for multiple testing in the gene-level GWEIS conducted in female subjects of UKB cohort on PHQ9 depression mean values in interaction with adult traumatic events scores, are marked with orange and their symbols. From the 11 inflammatory-related genes, which were significant in the GWEIS in female subjects of UKB cohort, genes with identical Z statistics' (positive) sign (n = 6) in the NM cohort are framed.

References

1. Garcia, F.J.; Sun, N.; Lee, H.; Godlewski, B.; Mathys, H.; Galani, K.; Zhou, B.; Jiang, X.; Ng, A.P.; Mantero, J.; et al. Single-cell dissection of the human brain vasculature. Nature 2022, 603, 893-899, doi:10.1038/s41586-022-04521-7.
2. Puvogel, S.; Alsema, A.; Kracht, L.; Webster, M.J.; Weickert, C.S.; Sommer, I.E.C.; Eggen, B.J.L. Single-nucleus RNA sequencing of midbrain blood-brain barrier cells in schizophrenia reveals subtle transcriptional changes with overall preservation of cellular proportions and phenotypes. Molecular Psychiatry 2022, 27, 4731-4740, doi:10.1038/s41380-022-01796-0.
